# Cervical Vestibular Evoked Myogenic Potentials in Benign Paroxysmal Positional Vertigo: A Systematic Review and Meta-Analysis

**DOI:** 10.3389/fneur.2019.01043

**Published:** 2019-10-01

**Authors:** Gang Chen, Gang Yu, Yun Li, Xuening Zhao, Xiaoyan Dai, Guotao Wang

**Affiliations:** Department of Otolaryngology Head and Neck Surgery, Shandong Provincial Hospital, Jinan, China

**Keywords:** benign paroxysmal positional vertigo, cervical vestibular evoked myogenic potentials, saccule, systematic review, meta-analysis

## Abstract

**Objective:** The objective of our study was to investigate the potential association between the occurrence of benign paroxysmal positional vertigo (BPPV) and saccular dysfunction using cervical vestibular evoked myogenic potentials (cVEMP) testing.

**Methods:** The databases including Pubmed, Embase, and CENTRAL were systemically searched for case-control literatures investigating saccular dysfunction using cVEMP testing in BPPV patients compared with healthy controls. The literatures were published up to 16 April 2019 and were limited to the English language. All statistical processes were carried out using software Review Manager, version 5.3. Subgroup analysis and sensitive analysis were performed simultaneously.

**Results:** Of the 12 case-control studies confirmed for meta-analysis, p13 latency of cVEMP was assessed in 8 studies, n23 latency in 6 studies, amplitude in 5 studies, asymmetry ratio (AR) in 3 studies, proportion of absent response in 9 studies, and abnormal cVEMP in 8 studies. Compared with healthy controls, the p13 mean latency of cVEMP was longer (MD = 0.88, 95% CI = 0.64–1.12, *p* < 0.00001), the mean amplitude was lower (SMD = −0.60, 95% CI = −0.80 to −0.41, *p* < 0.00001), and the proportions of absent response (OR = 8.76, 95% CI = 2.28–33.61, *p* = 0.002), and abnormal cVEMP (OR = 7.47, 95% CI = 4.65–12.01, *p* < 0.00001) were higher in BPPV patients. But there was no significant difference in the n23 mean latency (MD = 0.37, 95% CI = −0.23–0.98, *p* = 0.22) and the AR of cVEMP (MD = 3.95, 95% CI = −4.75–12.65, *p* = 0.37) between BPPV patients and healthy controls. In the sub-group analysis based on age, only the result of the proportion of absent response of cVEMP indicated a significant difference existed (*p* = 0.002) between the studies with age-matched controls (OR = 2.78, 95% CI = 1.09–7.10, *p* = 0.03) and the studies without age-matched controls (OR = 53.85, 95% CI = 10.09–287.13, *p* < 0.00001). In the sub-group analysis of the proportion of abnormal cVEMP according to the diagnostic criteria of abnormal cVEMP, the result indicated no significant difference existed between the four groups (*p* = 0.61, *I*^2^ = 0%). In the sensitivity analysis, we obtained the consistent results after removing each study sequentially.

**Conclusion:** The meta-analysis reveals that saccular dysfunction may be associated with BPPV occurrence, and neural degeneration in the saccular macula may be a potential pathogenesis for BPPV.

## Introduction

Vestibular evoked myogenic potentials (VEMPs) are short-latency and vestibule-dependent electromyographic (EMG) activities evoked by air-conducted loud sound (tone burst or click) (1), bone-conducted vibration (2), or galvanic stimuli (3). Generally, VEMPs contain cervical VEMPs and ocular VEMPs according to different effectors. Cervical VEMPs (cVEMPs), which were first described by Colebatch and Halmagyi (4), are biphasic surface potentials recorded from the sternocleidomastoid (SCM) muscles. They represent inhibition of the ipsilateral vestibulo-collic reflex, and reflect predominantly saccular and inferior vestibular nerve functions (5). Ocular VEMPs (oVEMPs) were found to be optimally recorded below the eyes opposite the stimulated ears, and to originate from the inferior oblique (IO) muscles by Rosengren et al. (6). Although there are still some controversies, oVEMPs may represent activation of the contralateral vestibulo-ocular reflex and reflect predominantly utricular and superior vestibular nerve functions (7). Consequently, VEMPs have become a popular evaluation measure of the otolith function over the past 20 years (8).

Benign paroxysmal positional vertigo (BPPV) is one of the most common peripheral vestibular diseases in specialist clinics of vertigo (9). It is characterized by the presence of brief and episodic vertigo or dizziness in response to head movement relative to gravity. Most BPPV cases having no clear etiology are divided into idiopathic BPPV. The pathogenesis of idiopathic BPPV is suspected to relate to degenerative process of the saccular or utricular macula (10). Based on the close anatomy relationship, utricular dysfunction is probably regarded as the pathogenesis of BPPV (11). However, the degenerative changes affect both the utricular and saccular maculae because of the anatomical and histological similarities between them (10). Otolith dysfunction derived from degenerative changes may cause the otoconia to detach from the saccular or utricular macula more easily (12).

So far many studies have investigated saccular dysfunction using cVEMP testing in BPPV patients compared with healthy controls, but the results are still inconsistent (10, 13–15). Furthermore, there are several parameters in cVEMP testing, such as latency, amplitude, asymmetry ratio (AR) and so on (16). Different studies used different parameters of cVEMP to analyze, so the conclusions of them were inevitably biased. So we systemically retrieved as many studies as possible and evaluated each parameter of cVEMP testing between BPPV patients and healthy controls. The objective of our study was to investigate the potential association between the BPPV occurrence and saccular dysfunction using cVEMP testing. As far as we know, this is the first systematic review and meta-analysis to investigate the cVEMP results in BPPV patients compared with healthy individuals.

## Materials and Methods

### Literature Search Strategy

**Figure 1:**
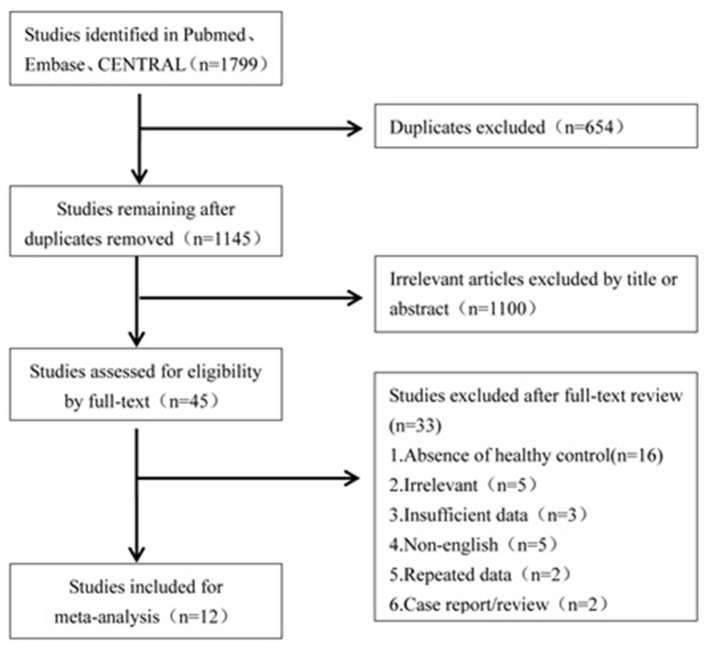
Flowchart of data search and studies selection for meta-analysis.

A literature search was conducted to retrieve all studies which investigated the cVEMP testing between BPPV patients and healthy controls. The databases including Pubmed, Embase, and CENTRAL were systemically searched for all relevant literatures. The literatures were published up to 16 April 2019 and were limited to the English language. The search strategies were “benign paroxysmal positional vertigo” and “vestibular evoked myogenic potential.” All the studies were sequentially examined through titles and abstracts screening, and full-text reading to identify studies to meta-analyze. All references of the included literatures were searched additionally. The flowchart is presented in Figure 1.

### Study Selection Criteria

Studies were included in the meta-analysis if they met the inclusion criteria: (1) retrospective or prospective case-control studies about cVEMP testing comparing BPPV patients with healthy controls; (2) diagnosis of BPPV relied on brief and recurrent vertigo and characteristic nystagmus in positional tests, such as Dix-Hallpike test and supine Roll test; (3) cVEMP outcomes in BPPV and healthy groups, such as latency, amplitude, AR, proportion of absent cVEMP, or proportion of abnormal cVEMP, were clearly stated in whole or in part; (4) healthy controls were subjects with normal hearing and no known history of vestibular and neurological disorders. If the data of literatures were duplicated, we selected the articles with the latest publication and complete data. The exclusion criteria were as follows: (1) case reports, reviews, comments, letters or practice guidelines; (2) absence of healthy controls; (3) unclear diagnosis of BPPV; (4) insufficient data of cVEMP to extract to compare BPPV patients with healthy controls; (5) BPPV patents with conductive hearing loss, or neurological diseases, or other otologic diseases (vestibular neuronitis, Meniere's disease, sudden sensorineural hearing loss, chronic otitis media, labyrinthitis, ototoxicity).

### Data Extraction

Data extraction and quantification were independently performed by two authors (GC and GY). Agreement on all the details was reached by discussion or by appealing to a third author. The following data were extracted from each study: first author, year of publication, country, research type, number of BPPV patients or healthy controls included, age, gender, number of each type of BPPV, number of included ears, type of acoustic stimuli, data of each parameter of cVEMP (p13 latency, n23 latency, p13-n23 peak to peak amplitudes, AR, number of ears with absent response, criteria for abnormal cVEMP, and number of ears with abnormal cVEMP). AR was calculated as 100[(Au–Aa)/(Au+Aa)], where Au is the p13-n23 amplitude on the unaffected side and Aa is the p13-n23 amplitude on the affected side.

### Quality Assessment

Two authors (GC and GY) performed the quality assessment according to the Newcastle-Ottawa Scale (NOS). Disagreement was resolved by appealing to a third author. Study quality was considered as high when the score had 6 or more stars (17).

### Statistical Analysis

All statistical processes were carried out using software Review Manager (RevMan), version 5.3. Mean difference (MD) and its 95% confidence interval (CI) were calculated to analyze p13 latency, n23 latency and AR, standardized mean difference (SMD) and its 95% CI were used to analyze peak to peak amplitude, and odds ratios (OR) and its 95% CI were used to analyze dichotomous variables between two groups. We used X2 and I^2^ index to evaluate the statistical heterogeneity in the meta-analysis. If *p* < 0.05 or *I*^2^ > 50%, the random-effects model was selected because of the significant heterogeneity, otherwise the fixed-effects model was selected. Considering the age may affect the results of cVEMP, sub-group analysis was performed based on whether or not the age matched between BPPV and healthy groups (18). So far, there has been no uniform diagnostic criterion for abnormal cVEMP. Then we performed a sub-group analysis according to the different diagnostic criteria of abnormal cVEMP. Publication bias of our meta-analysis was evaluated through funnel plots.

## Results

### Literature Screening

Of the 1,799 potentially relevant literatures, 654 studies were firstly removed for duplication. After screening titles and abstracts, 1,100 articles were excluded for irrelevance to our purpose. Of the remaining, 45 articles needed a full text screening, 16 were excluded for lack of healthy controls, 5 were excluded for irrelevance to our purpose, 5 were excluded for non-English publication, 2 were excluded for case report or reviews, and 2 were excluded because they came from the same team and the data may have an overlap with the third article. The third article with latest publication and complete data was then included (19). Finally, we confirmed 12 articles for meta-analysis (10, 13–15, 19–26).

### Characteristics of Studies Included

Twelve articles involved 537 BPPV patients and 489 healthy controls. Because bilateral BPPV cases were included in two studies (10, 14) and both ears of healthy controls in most studies were tested together, 545 ears in BPPV groups and 871 ears in healthy controls were included for cVEMP analysis. Of the 12 studies, 6 were prospective case-control studies (10, 15, 20, 21, 25, 26), and 8 included age-matched healthy controls (10, 13, 14, 19, 21–24). Eleven studies definitely excluded the individuals with conductive hearing loss in BPPV and control groups, except the study of Martínez Pascual et al. (20). cVEMP testing in all 12 studies were conducted through air-conducted sound. The characteristics of 12 included articles are described in Table 1. The detailed results of cVEMP testing for BPPV patients and healthy controls are shown in Table 2.

**Table 1:** The basic characteristics of all eligible studies.

**References**	**Country**	**Study type**	**Groups**	**No**.	**Gender(M:F)**	**Age(years) (mean ± SD)**	**Age matched**	**PSC- BPPV**	**HSC- BPPV**	**ASC- BPPV**	**Bilateral BPPV**	**MSC- BPPV**
Akkuzu et al. (10)	Turkey	Prospective	BPPV	25	6:19	52.9 ± 11.9	Yes	25	0	0	5	0
			Control	17	6:11	51.8 ± 15.8		–	–	–	–	–
Yang et al. (15)	Korea	Prospective	BPPV	41	12:29	Mean 59	No	34	7	0	0	0
			Control	92	NA	Mean 42		–	–	–	–	–
Korres et al. (14)	Greece	NA	BPPV	27	14:13	Median 45	Yes	27	0	0	3	0
			Control	30	17:13	Median 47		–	–	–	–	–
Longo et al. (25)	Italy	Prospective	BPPV	23	8:15	Mean 59	No	23	0	0	0	0
			Control	24	12:12	Mean 51		–	–	–	–	–
Eryaman et al. (26)	Turkey	Prospective	BPPV	31	12:19	51.9 ± 11.8	No	31	0	0	0	0
			Control	23	8:15	51.1 ± 10.8		–	–	–	–	–
Nakahara et al. (24)	Japan	NA	BPPV	12	5:7	Mean 65.5	Yes	12	0	0	0	0
			Control	12	6:6	Mean 63.1		–	–	–	–	–
Talaat et al. (23)	Egypt	NA	BPPV	112	52:60	46.2 ± 10.2	Yes	112	0	0	0	0
			Control	100	45:55	44.2 ± 9.9		–	–	–	–	–
Singh et al. (19)	India	NA	BPPV	31	NA	42 ± 5.7	Yes	31	0	0	0	0
			Control	31	NA	42.2 ± 5.8		–	–	–	–	–
Kim et al. (13)	Korea	NA	BPPV	102^*^	48:54	62.8 ± 13.1	Yes	47	51	0	0	4
			Control	50	23:27	60.1 ± 9.2		–	–	–	–	–
Karatas et al. (22)	Turkey	NA	BPPV	36	10:26	Mean 47.2	Yes	34	2	0	0	0
			Control	20	7:13	Mean 45.1		–	–	–	–	–
Xu et al. (21)	China	prospective	BPPV	30	12:18	Mean 45.5	Yes	30	0	0	0	0
			Control	30	10:20	Mean 42.2		–	–	–	–	–
Pascual et al. (20)	Spain	prospective	BPPV	67	16:51	Mean 58.06	No	67	0	0	0	0
			Control	60	23:37	Mean 46.3		–	–	–	–	–

* *Ten patients with bilateral BPPV were excluded*.

*No., number; M, male; F, female; NA, not available; SD, standard deviation; BPPV, benign paroxysmal positional vertigo; PSC, posterior semicircular canal; HSC, horizontal semicircular canal; ASC, anterior semicircular canal; MSC, multiple semicircular canal*.

**Table 2:** The detailed results of cervical vestibular evoked myogenic potentials (cVEMP) test.

**References**	**Groups**	**No. of included ears**	**Acoustic stimuli**	**Presence of response**					**Absent response**	**Abnormal cVEMP**	
				**No**.	**p13 latency (mean ± SD, ms)**	**n23 latency (mean ± SD, ms)**	**Amplitude (mean ± SD, uv)**	**cVEMP AR (mean ± SD,%)**	**NO**.	**Criteria**	**No**.
Akkuzu et al. (10)	BPPV	30	AC 500 Hz 100 dB nHL tone burst	30	14.3 ± 2.5	22.4 ± 2.4	39.8 ± 22.6	NA	0	1. p13 latency>15.7 ms or n23 latency>25.9 ms; 2. AR>59.7%; 3. NO response	9
	Control	34		34	13.7 ± 1.0	22.1 ± 1.9	57.9 ± 33.8	19.3 ± 20.2	0		2
Yang et al. (15)	BPPV	41	AC 95 dB clicks	30	14.99 ± 1.89	24.31 ± 2.26	NA	NA	11	NA	NA
	Control	184		184	13.25 ± 0.93	22.62 ± 1.76	NA	NA	0		NA
Korres et al. (14)	BPPV	30	AC 500 Hz 95 dB HL tone burst	27	17.30 ± 2.68	25.24 ± 2.87	NA	NA	3	1. p13 latency>19.50 ms or n23 latency>30.22 ms; 2. NO response	9
	Control	60		60	16.32 ± 1.59	24.62 ± 2.8	NA	NA	0		5
Longo et al. (25)	BPPV	23	AC 500 Hz 127 dB peSPL logon	18	14.79 ± 2.2	21.31 ± 1.81	16.01 ± 5.09	NA	5	1. p13 latency>17.09 ms or n23 latency>24.32 ms; 2. AR>36%; 3. NO response	9
	Control	48		48	14.27 ± 1.41	21.4 ± 1.46	17.78 ± 5.08	NA	0		2
Eryaman et al. (26)	BPPV	31	AC 500 Hz 100 dB nHL tone burst	24	15.75 ± 1.80	NA	NA	NA	7	1. p13 latency>17.21 ms; 2. NO response	12
	Control	60		60	14.95 ± 1.13	NA	NA	NA	0		0
NAkahara et al. (24)	BPPV	12	AC 500 Hz 125 dB SPL tone burst	NA	NA	NA	NA	NA	NA	1. p13 latency>17.7 ms or n23 latency>27.3 ms; 2. AR>41.6%; 3. NO response	3
	Control	24		NA	NA	NA	NA	NA	NA		4
Talaat et al. (23)	BPPV	112	AC 500 Hz 95 dB nHL tone burst	NA	NA	NA	NA	NA	NA	1. p13 latency>14.2 ms; 2. AR>32.6%; 3. NO response	15
	Control	200		200	12.6 ± 0.8	NA	NA	19.4 ± 6.3	0		1
Singh et al. (19)	BPPV	31	AC 500 Hz 125 dB SPL tone burst	30	16.27 ± 1.48	24.51 ± 0.94	5.86 ± 2.38^*^	15.68 ± 5.15	1	NA	NA
	Control	31		30	15.75 ± 0.78	24.67 ± 0.78	6.55 ± 2.35^*^	16.15 ± 4.47	1		NA
Kim et al. (13)	BPPV	102	AC 1,000 Hz 100 dB nHL tone burst	92	14.3 ± 1.5	NA	265.4 ± 165.3	22.1 ± 17.9	10	1. p13 latency>16.0 ms; 2. AR>25%; 3. NO response	29
	Control	100		94	13.2 ± 1.4	NA	395.6 ± 258.9	9.8 ± 7.6	6		NA
Karatas et al. (22)	BPPV	36	AC 500 Hz 100 dB nHL tone burst	36	14.2 ± 1.7	21.9 ± 2.0	0.6 ± 0.3^*^	16.6 ± 12.8	0	NA	NA
	Control	40		40	14.0 ± 1.3	21.8 ± 1.7	1.0 ± 0.4^*^	16.7 ± 13.4	0		NA
Xu et al. (21)	BPPV	30	AC 500 Hz 90 dB nHL tone burst	21	NA	NA	NA	NA	9	1. NO response	9
	Control	30		28	NA	NA	NA	NA	2		2
Pascual et al. (20)	BPPV	67	AC 500 Hz 100 dB tone burst	NA	NA	NA	NA	NA	NA	1. AR>33%; 2. NO response	33
	Control	60		NA	NA	NA	NA	NA	NA		10

* *cVEMP amplitudes were corrected using the background electromyographic activities of the sternocleidomastoid*.

*BPPV, benign paroxysmal positional vertigo; cVEMP, cervical vestibular evoked myogenic potentials; NA, not available; SD, standard deviation; No., number; AR, asymmetry ratio; AC, air-conducted; HL, hearing level; nHL, normal hearing level; SPL, sound pressure level; peSPL, peak equivalent sound pressure level*.

### Quality Assessment

We used NOS to assess the quality of the 12 eligible studies. The NOS scores ranged from 6 to 7 stars, and were all high-quality studies, as shown in Table 3. The main deduction items were the representativeness of the cases, selection of controls, and non-response rate.

**Table 3:** Quality assessment of the selected studies using the Newcastle-Ottawa Scale (NOS).

**References**	**Score on dimensions**			**Score**
	**Selection**	**Comparability**	**Exposure**	
Akkuzu et al. (10)	3	2	2	7
Yang et al. (15)	3	1	2	6
Korres et al. (14)	2	2	2	6
Longo et al. (25)	3	1	2	6
Eryaman et al. (26)	3	1	2	6
Nakahara et al. (24)	2	2	2	6
Talaat et al. (23)	3	2	2	7
Singh et al. (19)	2	2	2	6
Kim et al. (13)	3	2	2	7
Karatas et al. (22)	3	2	2	7
Xu et al. (21)	3	2	2	7
Pascual et al. (20)	3	1	2	6

### Meta-Analysis Results

#### P13 Latency of cVEMP in BPPV and Healthy Groups

**Figure 2:**
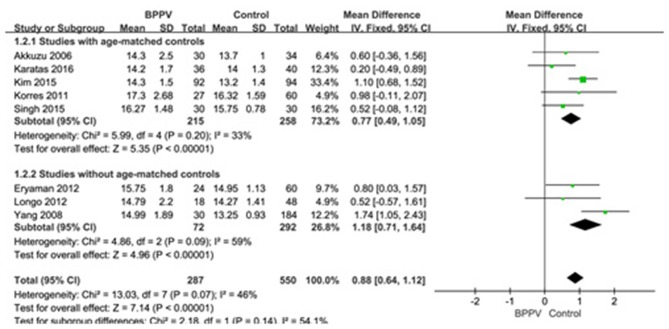
Forest plots of subgroup meta-analysis of p13 latency of cVEMP based on whether or not the age matches between BPPV and healthy groups. BPPV, benign paroxysmal positional vertigo; cVEMP, cervical vestibular evoked myogenic potential.

Eight studies (10, 13–15, 19, 22, 25, 26) compared mean p13 latency of cVEMP in BPPV and healthy groups. Because of no significant heterogeneity (*p* = 0.07, *I*^2^ = 46%, Figure 2), fixed-effects model was selected. The mean p13 latency of cVEMP in BPPV patients was significantly longer than that in healthy controls according to the forest plot (MD = 0.88, 95% CI = 0.64–1.12, *p* < 0.00001, Figure 2). In the sub-group analysis, no significant difference existed (*p* = 0.14, Figure 2) between the five studies with age-matched controls (MD = 0.77, 95% CI = 0.49–1.05, *p* < 0.00001, Figure 2) (10, 13, 14, 19, 22) and the three studies without age-matched controls (MD = 1.18, 95% CI = 0.71–1.64, *p* < 0.00001, Figure 2) (15, 25, 26).

#### N23 Latency of cVEMP in BPPV and Healthy Groups

**Figure 3:**
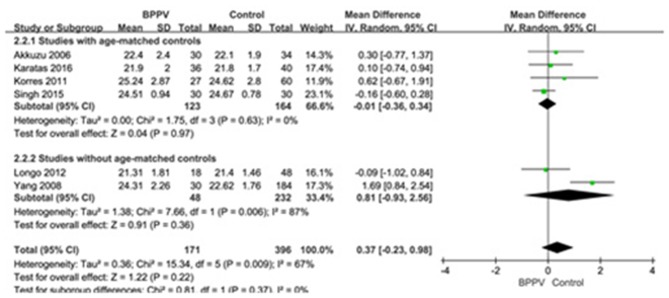
Forest plots of subgroup meta-analysis of n23 latency of cVEMP based on whether or not the age matches between BPPV and healthy groups. BPPV, benign paroxysmal positional vertigo; cVEMP, cervical vestibular evoked myogenic potential.

Six studies (10, 14, 15, 19, 22, 25) compared mean n23 latency of cVEMP in BPPV and healthy groups. Because of a significant heterogeneity (*p* = 0.009, *I*^2^ = 67%, Figure 3), random-effects model was selected. The mean n23 latency of cVEMP in BPPV patients was not different from that in healthy controls according to the forest plot (MD = 0.37, 95% CI = −0.23–0.98, *p* = 0.22, Figure 3). In the sub-group analysis, no significant difference existed (*p* = 0.37, Figure 3) between the four studies with age-matched controls (MD = −0.01, 95% CI = −0.36–0.34, *p* = 0.97, Figure 3) (10, 14, 19, 22) and two studies without age-matched controls (MD = 0.81, 95% CI = −0.93–2.56, Figure 3) (15, 25).

#### Amplitude of cVEMP in BPPV and Healthy Groups

**Figure 4:**
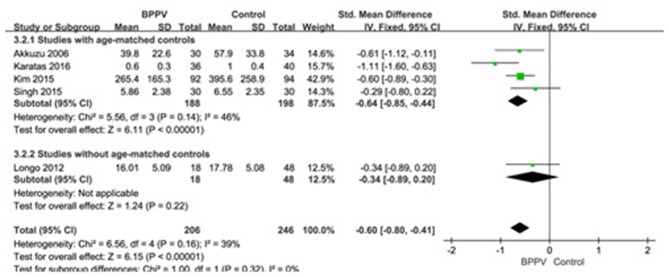
Forest plots of subgroup meta-analysis of peak to peak amplitude of cVEMP based on whether or not the age matches between BPPV and healthy groups. BPPV, benign paroxysmal positional vertigo; cVEMP, cervical vestibular evoked myogenic potential.

Five studies (10, 13, 19, 22, 25) assessed the peak to peak amplitude of cVEMP in BPPV and healthy groups. Because of no significant heterogeneity (*p* = 0.16, *I*^2^ = 39%, Figure 4), fixed-effects model was selected. The mean amplitude of cVEMP in BPPV patients was lower than that in healthy controls according to the forest plot (SMD = −0.60, 95% CI = −0.80 to −0.41, *p* < 0.00001, Figure 4). In the sub-group analysis, the result indicated that no significant difference existed (*p* = 0.32, Figure 4) between the four studies with age-matched controls (SMD = −0.64, 95% CI = −0.85 to −0.44, *p* < 0.00001, Figure 4) (10, 13, 19, 22) and one study without age-matched controls (SMD = −0.34, 95% CI = −0.89–0.20, *p* = 0.22, Figure 4) (25).

#### AR of cVEMP in BPPV and Healthy Groups

**Figure 5:**
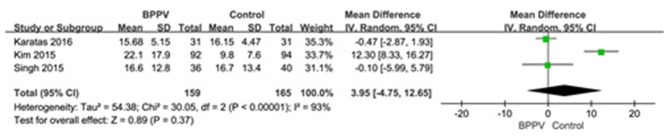
Forest plots of meta-analysis of asymmetry ratio (AR) of cVEMP between BPPV and healthy groups. BPPV, benign paroxysmal positional vertigo; cVEMP, cervical vestibular evoked myogenic potential.

Three studies (13, 19, 22) assessed the AR of cVEMP in BPPV and healthy groups. Because of a significant heterogeneity (*p* < 0.00001, *I*^2^ = 93%, Figure 5), random-effects model was selected. The AR of cVEMP in BPPV patients was not significantly different from that in healthy controls according to the forest plot (MD = 3.95, 95% CI = −4.75–12.65, *p* = 0.37, Figure 5). Considering all the three studies with age-matched control, no sub-group analysis was performed.

#### Proportion of Absent Response of cVEMP in BPPV and Healthy Groups

**Figure 6:**
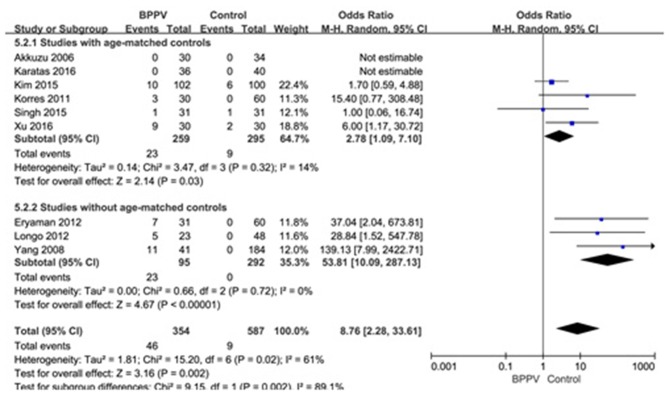
Forest plots of subgroup meta-analysis of proportion of absent response of cVEMP based on whether or not the age matches between BPPV and healthy groups. BPPV, benign paroxysmal positional vertigo; cVEMP, cervical vestibular evoked myogenic potential.

Nine studies (10, 13–15, 19, 21, 22, 25, 26) assessed the proportion of absent response of cVEMP in BPPV and healthy groups. Because of a significant heterogeneity (*p* = 0.02, *I*^2^ = 61%, Figure 6), random-effects model was selected. The proportion of absent response of cVEMP in BPPV patients was higher than that in healthy controls according to the forest plot (OR = 8.76, 95% CI = 2.28–33.61, *p* = 0.002, Figure 6). In the sub-group analysis, the result indicated a significant difference existed (*p* = 0.002, Figure 6) between the six studies with age-matched controls (OR = 2.78, 95% CI = 1.09–7.10, *p* = 0.03, Figure 6) (10, 13, 14, 19, 21, 22) and three studies without age-matched controls (OR = 53.85, 95% CI = 10.09–287.13, *p* < 0.00001, Figure 6) (15, 25, 26).

#### Proportion of Abnormal cVEMP in BPPV and Healthy Groups

**Figure 7:**
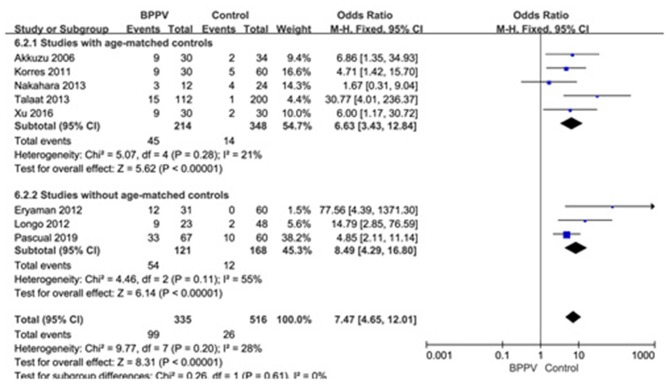
Forest plots of subgroup meta-analysis of proportion of abnormal cVEMP based on whether or not the age matches between BPPV and healthy groups. BPPV, benign paroxysmal positional vertigo; cVEMP, cervical vestibular evoked myogenic potential.

**Figure 8:**
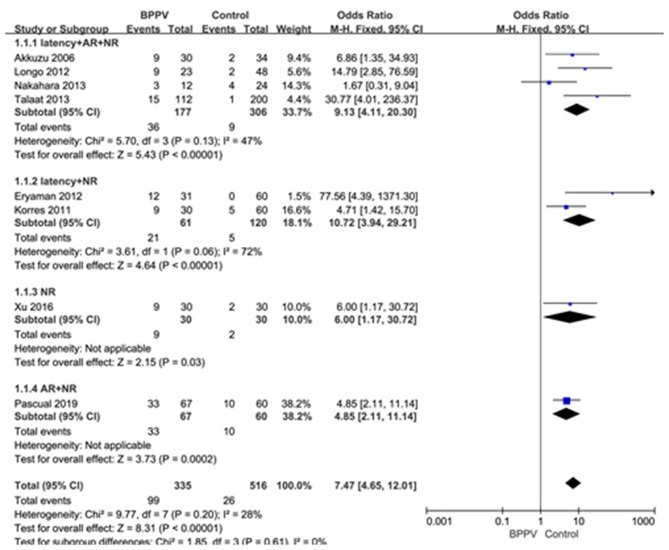
Forest plots of subgroup meta-analysis of proportion of abnormal cVEMP based on the different diagnostic criteria of abnormal cVEMP. BPPV, benign paroxysmal positional vertigo; cVEMP, cervical vestibular evoked myogenic potential; AR, asymmetry ratio; NR, no response.

Eight studies (10, 14, 20, 21, 23–26) assessed the proportion of abnormal cVEMP in BPPV and healthy groups. Because of no significant heterogeneity (*p* = 0.20, *I*^2^ = 28%, Figure 7), fixed-effects model was selected. The proportion of abnormal cVEMP in BPPV patients was higher than that in healthy controls according to the forest plot (OR = 7.47, 95% CI = 4.65–12.01, *p* < 0.00001, Figure 7). In the sub-group analysis based on age, the result indicated no significant difference existed (*p* = 0.61, Figure 7) between the five studies with age-matched controls (OR = 7.47, 95% CI = 4.65–12.01, *p* < 0.00001, Figure 7) (10, 14, 21, 23, 24) and three studies without age-matched controls (OR = 8.49, 95% CI = 4.29–16.80, *p* < 0.00001, Figure 7) (20, 25, 26). In the sub-group analysis according to the diagnostic criteria of abnormal cVEMP, the result indicated no significant difference existed between the four groups (*p* = 0.61, *I*^2^ = 0%, Figure 8).

#### Sensitivity Assessment and Publication Bias

**Figure 9:**
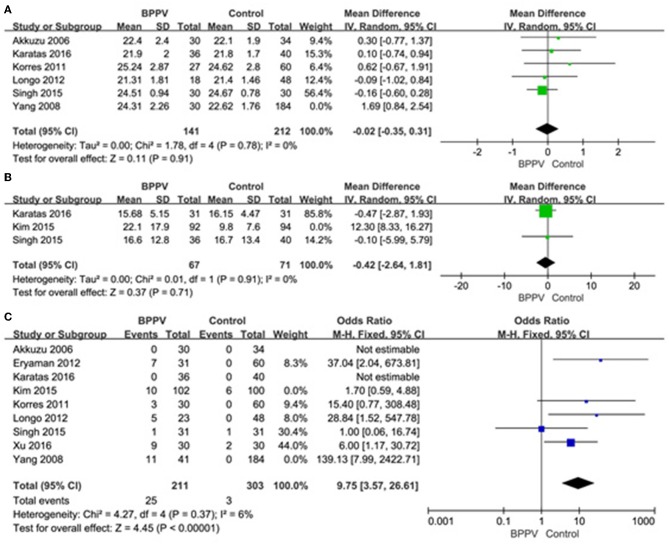
**(A)** Sensitive analysis of n23 latency of cVEMP, indicating that the study of Yang et al. is the source of high heterogeneity. **(B)** Sensitive analysis of asymmetry ratio (AR) of cVEMP, indicating that the study of Kim et al. is the source of high heterogeneity. **(C)** Sensitive analysis of proportion of absent response of cVEMP, indicating that the studies of Yang et al. and Kim et al. are the source of high heterogeneity. BPPV, benign paroxysmal positional vertigo; cVEMP, cervical vestibular evoked myogenic potential.

**Figure 10:**
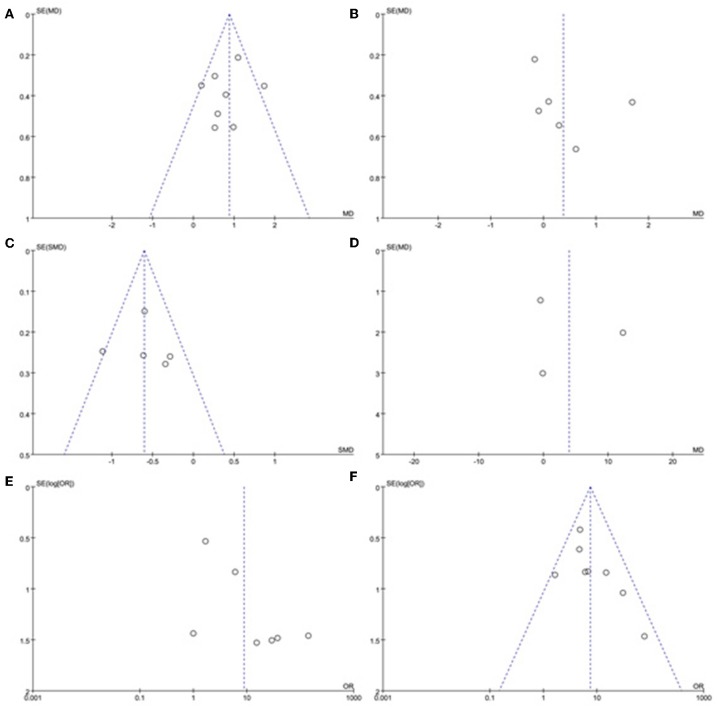
Funnel plots for the evaluation of publication bias in the selected studies. **(A)** Funnel plots of p13 latency of cVEMP. **(B)** Funnel plots of n23 latency of cVEMP. **(C)** Funnel plots of amplitude of cVEMP. **(D)** Funnel plots of p13 asymmetry ratio of cVEMP. **(E)** Funnel plots of proportion of absent response of cVEMP. **(F)** Funnel plots of proportion of abnormal cVEMP. BPPV, benign paroxysmal positional vertigo; cVEMP, cervical vestibular evoked myogenic potential.

A sensitivity analysis was performed in each meta-analysis. After removing each study sequentially to evaluate the reliability of our conclusions, we obtained the consistent results (Supplementary Figures). However, after removal of the study of Yang et al. (15), the heterogeneity became insignificant in the meta-analysis of n23 latency (*p* = 0.78, *I*^2^ = 0%, Figure 9A). After removal of the study of Kim et al. (13), the heterogeneity became insignificant in the meta-analysis of AR (*p* = 0.91, *I*^2^ = 0%, Figure 9B). After removal of the studies of Yang et al. (15) and Kim et al. (13), the heterogeneity became insignificant in the meta-analysis of the proportion of absent response of cVEMP (*p* = 0.37, *I*^2^ = 6%, Figure 9C). These suggested that the source of high heterogeneity in the three meta-analyses above were from study of Yang et al. (15) or/and the study of Kim et al. (13). Asymmetry was observed in the pool of data from the included literatures, and publication bias was obviously indicated, as shown by funnel plots (Figure 10).

## Discussion

The pathophysiology of BPPV remains unclear, and it is suspected that otolith debris detaches from macula of the otolith membrane, and migrates into the endolymphatic lumen of semicircular canals (27) or attaches to the cupula of the semicircular canals (28). The cause of dislodged otoconia in secondary BPPV may be attributed to head trauma (29) or inner ear diseases such as sudden sensorineural hearing loss (30) and Meniere's disease (31). But otolithic degenerative changes related to age (32) and osteoporosis (33) and so on, may be responsible for the occurrence of idiopathic BPPV. Due to the similarities in anatomy and histology, the degenerative progress not only affects the utricular macula, but also injures saccular macula which can be detected by cVEMP.

Considerable previous studies have reported that there were higher incidences of abnormal cVEMP in the BPPV groups compared with the healthy controls (13, 15, 26). But so far there has been a controversy about this argument (14, 22, 24). Akkuzu et al. (10) and Korres et al. (14) found that the mean latency of p13 and n23 of cVEMP in BPPV cases was not significantly different from that in healthy individuals. Nakahara et al. (24) reported that the proportion of abnormal cVEMP indicated no significant difference between the two groups. As far as we know, this is the first systematic review and meta-analysis to investigate the cVEMP results in BPPV patients compared with healthy individuals based on 12 case-control studies with high-quality, and to explore possible association between BPPV occurrence and saccular dysfunction.

In our meta-analysis, we first confirmed that the mean p13 latency was significantly longer in the affected ears of BPPV groups than that in healthy controls, but the difference in mean n23 latency between the two groups was statistically insignificant. Gacek et al. (34) performed post-mortem examinations of temporal bones from five BPPV patients and found the loss of vestibular ganglion cells in the inferior vestibular nerve, and neuron degeneration in the saccular nerve which was a part of inferior vestibular nerve. The delay of p13 latency may be associated with the neural degeneration of the saccular nerve. The mechanism has not been understood clearly. We speculate that the disorder of myelin sheath of the saccular nerve which was induced by the degeneration process may be responsible for the delay of p13 latency through reducing nerve conduction velocity (35, 36). Besides, prolonged latency of p13 or n23 might indicate the injury of the cVEMP reflex pathway. Retro-labyrinthine lesions, including large cerebellopontine angle tumors, or central disorders including multiple sclerosis, were also responsible for the prolongation of p13 latency mainly due to the vestibulospinal tract lesions (37). Considering that age may affect the latency of p13 and n23 of cVEMP, we performed a sub-group analysis, but the differences were insignificant according to our results (*p* = 0.14 for p13 latency and *p* = 0.37 for n23 latency). Therefore, regardless of the age, p13 latency was delayed in BPPV patients but n23 latency was not affected.

Secondly, our analysis also confirmed that the mean amplitude in the affected ears of BPPV groups was significantly lower than that in healthy controls. Because of the low reproducibility and large variation of amplitude of cVEMP, only five studies assessed the difference between the two groups. Two of the studies corrected the amplitude using the background EMG activity (19, 22), then we used SMD and its 95% CI to meta-analyse amplitude, and found that the difference of mean amplitude between BPPV groups and healthy controls was significant with no significant heterogeneity. Considering the age may affect the amplitude of cVEMP, we also performed a sub-group analysis, but the differences were insignificant according our results (*p* = 0.32). This may be due to only one study without age-matched controls being included. In addition, the difference in AR of cVEMP between the two groups was statistically insignificant, but only three studies were enrolled to meta-analyse with a significant heterogeneity. Kataras et al. (22) found that the amplitudes from the affected and unaffected ears of unilateral BPPV patients were similar, suggesting bilateral involvement of neural degeneration in unilateral BPPV. This may partly explain the insignificant difference of AR in BPPV groups compared with healthy controls.

Thirdly, we also found that the proportion of absent response of cVEMP in the affected ears in BPPV groups was significantly higher than that in healthy controls. The proportion of absent response of cVEMP in the affected ears of BPPV groups ranged from 0 to 30% (10, 21, 22) in included studies, resulting in a significant heterogeneity. According to sensitivity analysis, we found the heterogeneity was from the studies of Kim et al. (13) and Yang et al. (15). In the study of Yang et al. (15), up to 26.8% of affected ears in BPPV groups showed no response in cVEMP while both ears of all individuals in healthy controls showed a response in cVEMP. In the study of Kim et al. (13), the sample size was large and only 9.8% of affected ears in BPPV groups showed no response of cVEMP. The occurrence of absent response usually indicated that the degeneration of saccular macula was extensive (26). In addition, conductive hearing loss may cause the absence of cVEMP response. Only the study of Martínez Pascual et al. (20) in our included articles did not definitely emphasize the exclusion of conductive hearing loss, but it was not the source of heterogeneity according to sensitivity analysis. Considering the age may affect the proportion of absent response, we performed a sub-group analysis, and the differences were significant according to our results (*p* = 0.002) with high heterogeneity (*I*^2^ = 89.1%). So age might be an important factor for analysis of proportion of absent response of cVEMP.

Lastly, we meta-analyzed the proportion of abnormal cVEMP based on their own criteria for abnormal cVEMP in eight studies included, and found that the proportion of abnormal cVEMP in the affected ears of BPPV groups was significantly higher than that in healthy controls. We performed a sub-group analysis based on age, and the differences were insignificant between studies with and without age-matched controls (p = 0.61). The proportion of abnormal cVEMP ranged from 13.4 to 49.2% (20, 23) in our studies included in meta-analysis, which varied rather largely. This was mainly due to diverse criteria for defining abnormality of cVEMP. Then we performed sub-group analysis according to the diagnostic criteria of abnormal cVEMP, and the result indicated no significant difference existed between the four groups. Even so, we should constitute uniform criteria as soon as possible and conduct further studies adopting uniform criteria.

There are still a few limitations to be considered in our meta-analysis. First of all, the sample size of our meta-analysis was not large enough, and all the included articles compared BPPV patients with healthy controls using different parameters of cVEMP. Secondly, all included articles were case-control studies and lack of randomized control trials. Thirdly, the different criteria for defining abnormality of every parameters of cVEMP and some studies without age-matched control existed, probably resulting in heterogeneity. In the future, well-designed prospective case-control studies with age-matched controls and uniform criteria of cVEMP testing should be conducted to investigate the saccular dysfunction compared BPPV patients with healthy controls.

## Conclusion

In spite of some shortcomings, we have given a credible conclusion that there are several distinctive characteristics of cVEMP testing in the BPPV patients compared with healthy controls, including longer latency of p13, lower amplitude of p13-n23, and higher proportion of absent response. It is inferred that abnormality of cVEMP may be associated with BPPV occurrence, and neural degeneration in the saccular macula may be a potential pathogenesis for BPPV.

## Author Contributions

GC and GY contributed to the study design, statistical analysis, and manuscript draft. All authors helped to perform the analysis and to revise the manuscript with constructive discussions.

### Conflict of Interest

The authors declare that the research was conducted in the absence of any commercial or financial relationships that could be construed as a potential conflict of interest.
