# Supplementary material for: Cervical Vestibular Evoked Myogenic Potentials in Benign Paroxysmal Positional Vertigo: A Systematic Review and Meta-Analysis
Source: Front Neurol. 2019 Oct 1;10:1043. doi: 10.3389/fneur.2019.01043 (PMC6779767; doi:10.3389/fneur.2019.01043)
Supplement: Supplementary file 1 [file Data_Sheet_1.docx]

Supplement figure1.Sensitive analysis of p13 latency of cVEMP by removing the study of Akkuzu et al.

Supplement figure 2.Sensitive analysis of p13 latency of cVEMP by removing the study of Eryaman et al.

Supplement figure 3.Sensitive analysis of p13 latency of cVEMP by removing the study of Karatas et al.

Supplement figure 4.Sensitive analysis of p13 latency of cVEMP by removing the study of Kim et al.

Supplement figure 5.Sensitive analysis of p13 latency of cVEMP by removing the study of Korres et al.

Supplement figure 6.Sensitive analysis of p13 latency of cVEMP by removing the study of Longo et al.

Supplement figure 7.Sensitive analysis of p13 latency of cVEMP by removing the study of Singh et al.

Supplement figure 8.Sensitive analysis of p13 latency of cVEMP by removing the study of Yang et al.

Supplement figure 9.Sensitive analysis of n23 latency of cVEMP by removing the study of Akkuzu et al.

Supplement figure 10.Sensitive analysis of n23 latency of cVEMP by removing the study of Karatas et al.

Supplement figure 11.Sensitive analysis of n23 latency of cVEMP by removing the study of Korres et al.

Supplement figure 12.Sensitive analysis of n23 latency of cVEMP by removing the study of Longo et al.

Supplement figure 13.Sensitive analysis of n23 latency of cVEMP by removing the study of Singh et al.

Supplement figure 14.Sensitive analysis of n23 latency of cVEMP by removing the study of Yang et al.

Supplement figure 15.Sensitive analysis of peak to peak amplitude of cVEMP by removing the study of Akkuzu et al.

Supplement figure 16.Sensitive analysis of peak to peak amplitude of cVEMP by removing the study of Karatas et al.

Supplement figure 17.Sensitive analysis of peak to peak amplitude of cVEMP by removing the study of Kim et al.

Supplement figure 18.Sensitive analysis of peak to peak amplitude of cVEMP by removing the study of Longo et al.

Supplement figure 19.Sensitive analysis of peak to peak amplitude of cVEMP by removing the study of Singh et al.

Supplement figure 20.Sensitive analysis of asymmetry ratio (AR) of cVEMP by removing the study of Karatas et al.

Supplement figure 21.Sensitive analysis of asymmetry ratio (AR) of cVEMP by removing the study of Kim et al.

Supplement figure 22.Sensitive analysis of asymmetry ratio (AR) of cVEMP by removing the study of Singh et al.

Supplement figure 23.Sensitive analysis of proportion of absent response of cVEMP by removing the study of Akkuzu et al.

Supplement figure 24.Sensitive analysis of proportion of absent response of cVEMP by removing the study of Eryaman et al.

Supplement figure 25.Sensitive analysis of proportion of absent response of cVEMP by removing the study of Karatas et al.

Supplement figure 26.Sensitive analysis of proportion of absent response of cVEMP by removing the study of Kim et al.

Supplement figure 27.Sensitive analysis of proportion of absent response of cVEMP by removing the study of Korres et al.

Supplement figure 28.Sensitive analysis of proportion of absent response of cVEMP by removing the study of Longo et al.

Supplement figure 29.Sensitive analysis of proportion of absent response of cVEMP by removing the study of Singh et al.

Supplement figure 30.Sensitive analysis of proportion of absent response of cVEMP by removing the study of Xu et al.

Supplement figure 31.Sensitive analysis of proportion of absent response of cVEMP by removing the study of Yang et al.

Supplement figure 32.Sensitive analysis of proportion of absent response of cVEMP by removing the study of Kim and Yang et al.

Supplement figure 33.Sensitive analysis of proportion of abnormal cVEMP by removing the study of Akkuzu et al.

Supplement figure 34.Sensitive analysis of proportion of abnormal cVEMP by removing the study of Eryaman et al.

Supplement figure 35.Sensitive analysis of proportion of abnormal cVEMP by removing the study of Korres et al.

Supplement figure 36.Sensitive analysis of proportion of abnormal cVEMP by removing the study of Longo et al.

Supplement figure 37.Sensitive analysis of proportion of abnormal cVEMP by removing the study of Nakahara et al.

Supplement figure 38.Sensitive analysis of proportion of abnormal cVEMP by removing the study of Pascual et al.

Supplement figure 39.Sensitive analysis of proportion of abnormal cVEMP by removing the study of Talaat et al.

Supplement figure 40.Sensitive analysis of proportion of abnormal cVEMP by removing the study of Xu et al.
