# Supplementary figures and images for: Cervical Vestibular Evoked Myogenic Potentials in Benign Paroxysmal Positional Vertigo: A Systematic Review and Meta-Analysis
Source: Front Neurol. 2019 Oct 1;10:1043. doi: 10.3389/fneur.2019.01043 (PMC6779767; doi:10.3389/fneur.2019.01043)

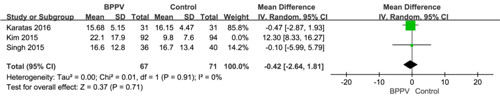

Supplement: Supplementary file 2 [file Data_Sheet_2.zip › supplement figures/Supplement figure 22.jpg]

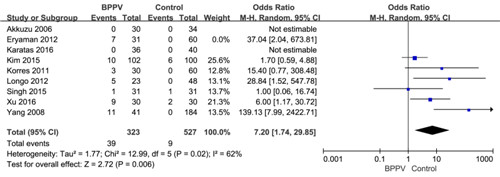

Supplement: Supplementary file 2 [file Data_Sheet_2.zip › supplement figures/Supplement figure 24.jpg]

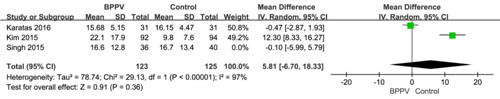

Supplement: Supplementary file 2 [file Data_Sheet_2.zip › supplement figures/Supplement figure 20.jpg]

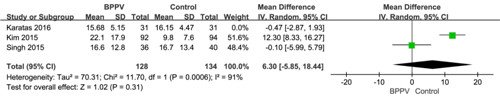

Supplement: Supplementary file 2 [file Data_Sheet_2.zip › supplement figures/Supplement figure 21.jpg]

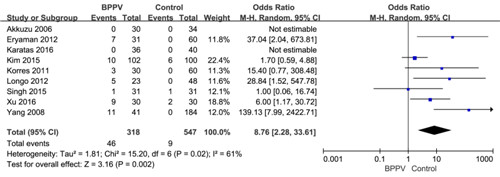

Supplement: Supplementary file 2 [file Data_Sheet_2.zip › supplement figures/Supplement figure 25.jpg]

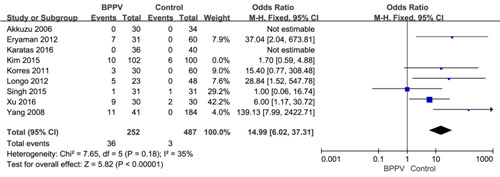

Supplement: Supplementary file 2 [file Data_Sheet_2.zip › supplement figures/Supplement figure 26.jpg]

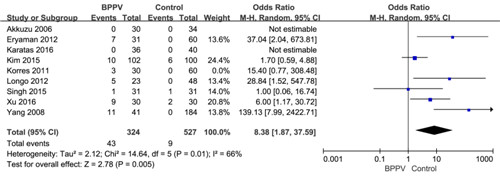

Supplement: Supplementary file 2 [file Data_Sheet_2.zip › supplement figures/Supplement figure 27.jpg]

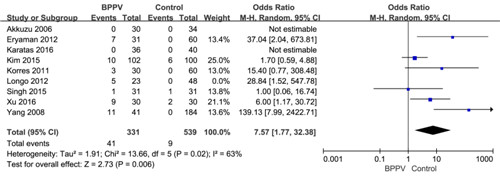

Supplement: Supplementary file 2 [file Data_Sheet_2.zip › supplement figures/Supplement figure 28.jpg]

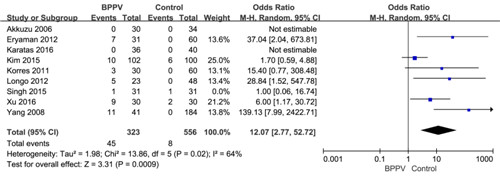

Supplement: Supplementary file 2 [file Data_Sheet_2.zip › supplement figures/Supplement figure 29.jpg]

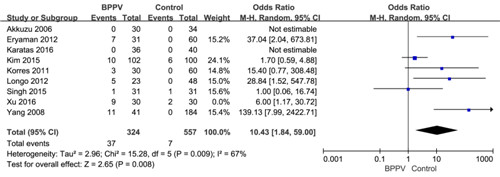

Supplement: Supplementary file 2 [file Data_Sheet_2.zip › supplement figures/Supplement figure 30.jpg]

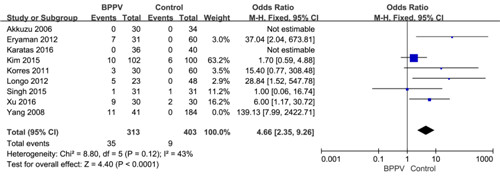

Supplement: Supplementary file 2 [file Data_Sheet_2.zip › supplement figures/Supplement figure 31.jpg]

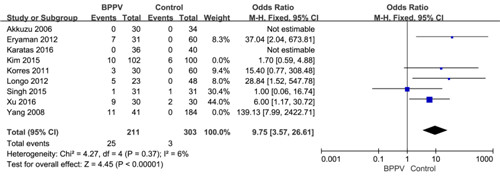

Supplement: Supplementary file 2 [file Data_Sheet_2.zip › supplement figures/Supplement figure 32.jpg]

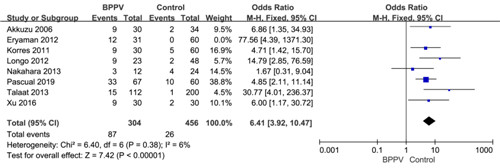

Supplement: Supplementary file 2 [file Data_Sheet_2.zip › supplement figures/Supplement figure 34.jpg]

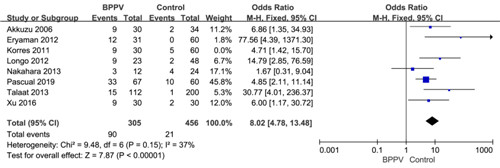

Supplement: Supplementary file 2 [file Data_Sheet_2.zip › supplement figures/Supplement figure 35.jpg]

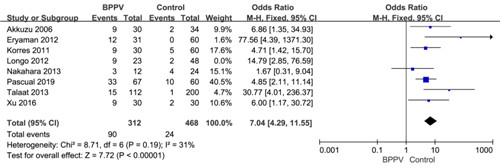

Supplement: Supplementary file 2 [file Data_Sheet_2.zip › supplement figures/Supplement figure 36.jpg]

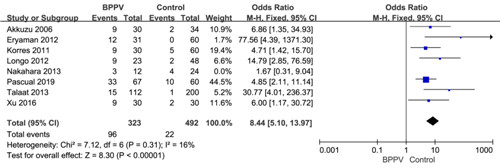

Supplement: Supplementary file 2 [file Data_Sheet_2.zip › supplement figures/Supplement figure 37.jpg]

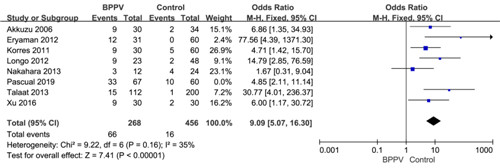

Supplement: Supplementary file 2 [file Data_Sheet_2.zip › supplement figures/Supplement figure 38.jpg]

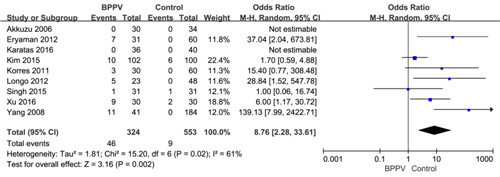

Supplement: Supplementary file 2 [file Data_Sheet_2.zip › supplement figures/Supplement figure 23.jpg]

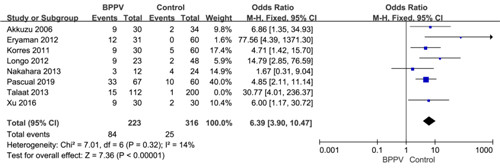

Supplement: Supplementary file 2 [file Data_Sheet_2.zip › supplement figures/Supplement figure 39.jpg]

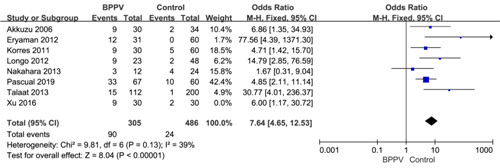

Supplement: Supplementary file 2 [file Data_Sheet_2.zip › supplement figures/Supplement figure 40.jpg]

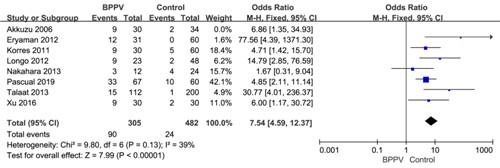

Supplement: Supplementary file 2 [file Data_Sheet_2.zip › supplement figures/Supplement figure 33.jpg]

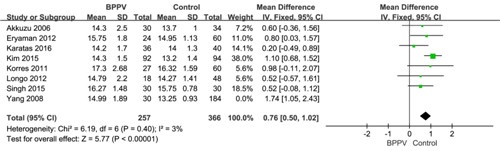

Supplement: Supplementary file 2 [file Data_Sheet_2.zip › supplement figures/Supplement figure 8.jpg]

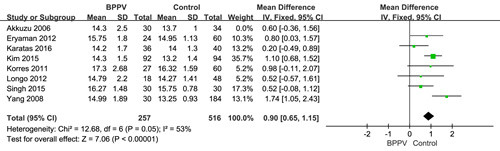

Supplement: Supplementary file 2 [file Data_Sheet_2.zip › supplement figures/Supplement figure 1 .jpg]

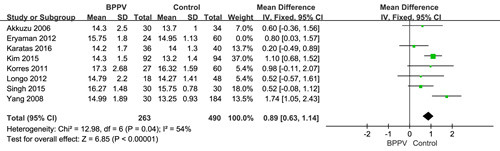

Supplement: Supplementary file 2 [file Data_Sheet_2.zip › supplement figures/Supplement figure 2.jpg]

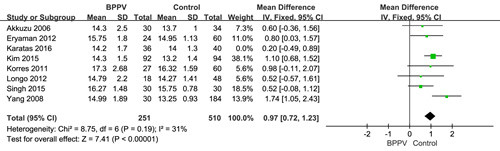

Supplement: Supplementary file 2 [file Data_Sheet_2.zip › supplement figures/Supplement figure 3.jpg]

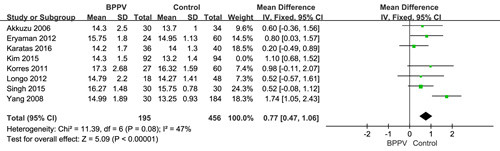

Supplement: Supplementary file 2 [file Data_Sheet_2.zip › supplement figures/Supplement figure 4.jpg]

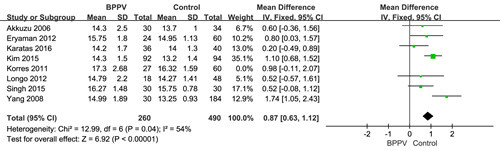

Supplement: Supplementary file 2 [file Data_Sheet_2.zip › supplement figures/Supplement figure 5.jpg]

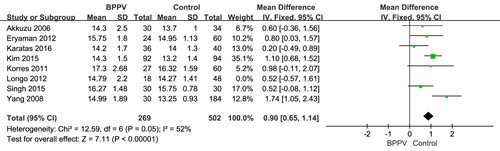

Supplement: Supplementary file 2 [file Data_Sheet_2.zip › supplement figures/Supplement figure 6.jpg]

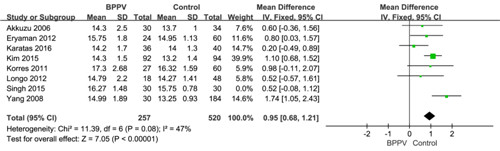

Supplement: Supplementary file 2 [file Data_Sheet_2.zip › supplement figures/Supplement figure 7.jpg]

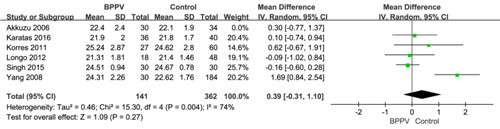

Supplement: Supplementary file 2 [file Data_Sheet_2.zip › supplement figures/Supplement figure 9.jpg]

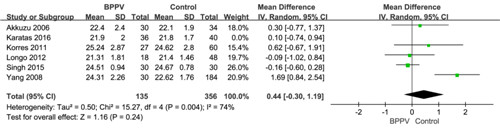

Supplement: Supplementary file 2 [file Data_Sheet_2.zip › supplement figures/Supplement figure 10.jpg]

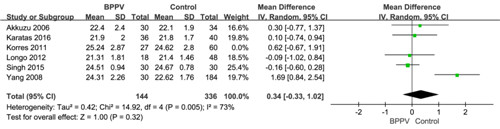

Supplement: Supplementary file 2 [file Data_Sheet_2.zip › supplement figures/Supplement figure 11.jpg]

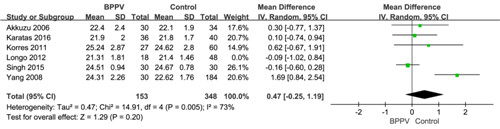

Supplement: Supplementary file 2 [file Data_Sheet_2.zip › supplement figures/Supplement figure 12.jpg]

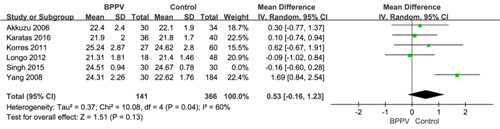

Supplement: Supplementary file 2 [file Data_Sheet_2.zip › supplement figures/Supplement figure 13.jpg]

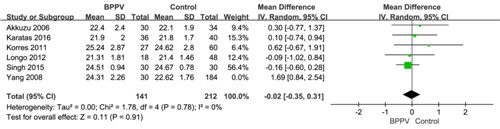

Supplement: Supplementary file 2 [file Data_Sheet_2.zip › supplement figures/Supplement figure 14.jpg]

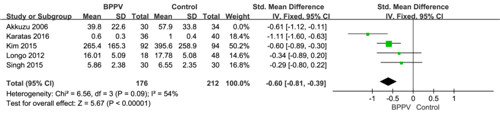

Supplement: Supplementary file 2 [file Data_Sheet_2.zip › supplement figures/Supplement figure 15.jpg]

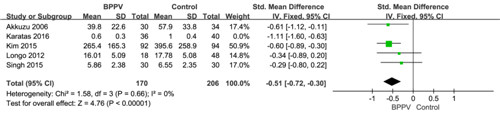

Supplement: Supplementary file 2 [file Data_Sheet_2.zip › supplement figures/Supplement figure 16.jpg]

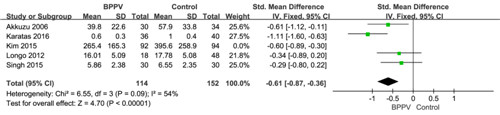

Supplement: Supplementary file 2 [file Data_Sheet_2.zip › supplement figures/Supplement figure 17.jpg]

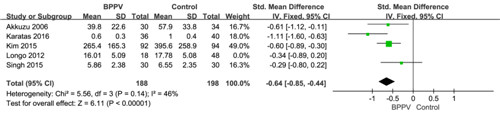

Supplement: Supplementary file 2 [file Data_Sheet_2.zip › supplement figures/Supplement figure 18.jpg]

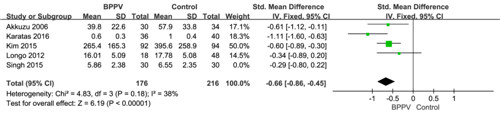

Supplement: Supplementary file 2 [file Data_Sheet_2.zip › supplement figures/Supplement figure 19.jpg]
